# Evolutive acid-base derangements in critically ill patients: epidemiological aspects, association with mortality and metabolic acidosis prediction

**DOI:** 10.62675/2965-2774.20260160

**Published:** 2026-01-09

**Authors:** Carine Carrijo de Faria, Caterina Lure Nema Paiva, Luiz Marcelo Almeida de Araujo, Luis Carlos Maia Cardozo, Marcelo Park

**Affiliations:** 1 Universidade de São Paulo Faculdade de Medicina Hospital das Clínicas São Paulo SP Brazil Intensive Care Unit, Hospital das Clínicas, Faculdade de Medicina, Universidade de São Paulo - São Paulo (SP), Brazil.

**Keywords:** Acid-base imbalance, Metabolic acidosis, Acidosis, respiratory, Alkalosis, respiratory, Mortality, Critical illness, Prevalence, Alkalosis, Hospitalization, Intensive care units

## Abstract

**Objective:**

To assess the prevalence and evolution of acid-base disturbances at intensive care unit admission and throughout hospitalization and their association with intensive care unit mortality.

**Methods:**

A retrospective epidemiological study was conducted, analyzing consecutive patients admitted to a single intensive care unit.

**Results:**

Metabolic acidosis, either isolated or combined with other disturbances, was the most prevalent disorder (58.0%), followed by respiratory alkalosis (37.6%), respiratory acidosis (25.7%), and metabolic alkalosis (12.8%). Multivariate analysis demonstrated that metabolic alkalosis combined with respiratory alkalosis was independently associated with reduced mortality (OR 0.427, 95%CI 0.194 - 0.869). Higher standard base excess at intensive care unit admission was correlated with lower mortality (OR 0.973, 95%CI 0.956 - 0.990). Maximum pCO_2_ variation during hospitalization showed no significant association with mortality. However, greater standard base excess improvement was independently linked to reduced mortality in patients with intensive care unit stays exceeding 5 days. Additionally, disease severity markers and younger age were predictive of metabolic acidosis both at admission and during hospitalization.

**Conclusion:**

Metabolic acidosis was the most common acid-base disorder at intensive care unit admission, with lower standard base excess levels associated with increased mortality. Standard base excess improvement during prolonged intensive care unit stays correlated with improved survival. Disease severity indicators were predictive of metabolic acidosis upon admission and throughout hospitalization.

## INTRODUCTION

Acid-base derangements are frequent in critically ill patients.^(1,2)^ Metabolic acidosis has a well-established association with intensive care unit (ICU) outcomes,^(3,4)^ especially when secondary to unmeasured anions^(5)^ and lactate^(6)^ elevations. It is still unknown whether this association is causal.^(7)^ However, from a physiological perspective, acid-base status regulates intracellular function, and its equilibrium is essential to maintain homeostasis.^(8)^

Despite the great clinical relevance of critically ill patients’ bedside knowledge about acid-base disturbances, there are some gaps in the current literature: the characterization of acid-base epidemiology is scarce and incredibly variable, depending on the case mix;^(9–11)^ the association of respiratory disturbances with ICU outcomes is not clear;^(12)^ the epidemiology of the changes in acid-base status after ICU admission, as well as their association with outcomes, is not well explored.

Based on the current acid-base literature uncertainties, we conducted a retrospective analysis of a retrospective cohort of critically ill adult patients admitted to a general intensive care unit. The aim of the study was to assess the prevalence and evolution of acid-base disturbances at intensive care unit admission and throughout hospitalization and their association with intensive care unit mortality.

## METHODS

We retrieved the data from a prospectively collected database of a general ICU in *Hospital das Clínicas* of the *Universidade de São Paulo*, in Brazil. The Informed Consent was waived by the Research Ethics Committee of *Hospital das Clínicas* of the *Universidade de São Paulo*, due to its observational nature (registration number 107.443).

### Patients, hospital, and intensive care unit

We enrolled all consecutive patients admitted to the ICU from March 15, 2007 to September 11, 2019. *Hospital das Clínicas* complex is a public university-affiliated hospital funded by the Brazilian Unified Health System (*Sistema Único de Saúde* - SUS) that has 2,500 beds and 450 ICU-beds. This study comprises patients admitted to the mixed-ICU. Due to variable funding, the ICU currently has 12 beds, but ranged from 5 to 12 beds during the study period.

### General intensive care unit care

The ICU is operated 24 hours / 7 weekdays by 1 nurse / 6 beds, 1 nurse-technician / 2 beds, 1 physiotherapist / 10 beds, and 1 senior intensivist, in accordance with Brazilian Health Ministry recommendations. Additionally, six medical and two physiotherapy residents work in the ICU during the day, and one medical resident works at night. The ICU offers complex acute care support, such as continuous renal replacement therapy, plasmapheresis, intracranial pressure monitoring, respiratory and cardiac extracorporeal membrane oxygenation, as well as complex rehabilitation, including persistent mechanical ventilation (MV) weaning, motor deficits in strokes, Guillain-Barré syndrome, and Myasthenia Gravis.

We follow the current best evidence-based practices recommended for critically ill patients. Additional care details were already published.^(13)^

### Data collection

Two senior intensivists prospectively collected daily data in a local database hosted by Microsoft Access^®^. The dataset comprises epidemiological data, daily physiological data, sequential organ failure assessment, laboratory data, MV parameters, and the use of invasive devices, vasoactive drugs, sedation, and feeding. Daily blood gas analyses were collected until death or ICU discharge. Acid-base variables were obtained preferably from arterial blood gas analysis. When unavailable, venous samples were used. Patients were followed up until ICU discharge.

### Definitions

- Physiological data: the admission physiological data are the lowest and highest values of the vital signs (heart rate, respiratory rate, arterial pressure, and body temperature) during the first 12 hours after ICU admission.
- Modified Charlson score:^(14)^ the Charlson score was used to quantify the comorbidity burden of each patient and was partially calculated using age, myocardial infarction, heart failure, stroke, transitory ischemic attack, chronic obstructive pulmonary disease (COPD), cirrhosis, diabetes mellitus, dialysis, Acquired Immunodeficiency Syndrome (AIDS), and neoplasms. The maximum points using this modified score are 27.
- Syndromic diagnoses: the syndromic diagnoses were categorized according to the Simplified Acute Physiological Score (SAPS) 3 definitions.^(15,16)^
- Acid-base diagnoses: we classified the acid-base diagnoses according to the pH as acidemia, normal, or alkalemia, according to the partial pressure of carbon dioxide (pCO_2_) as respiratory acidosis, normal, or respiratory alkalosis, and according to the standard base excess (SBE) as metabolic acidosis, normal, or metabolic alkalosis. The thresholds were:
  1. pH < 7.35 was considered acidemia, and pH > 7.45 was considered alkalemia.^(17,18)^
  2. pCO_2_ < 35mmHg was considered respiratory alkalosis, and pCO_2_ > 45mmHg was considered respiratory acidosis.^(17,18)^
  3. SBE < - 2mEq/L was considered metabolic acidosis, and SBE > + 2mEq/L was considered metabolic alkalosis.^(19–21)^

### Data presentation and statistics

Quantitative data are presented as mean ± standard deviation, and qualitative data as the number of occurrences and percentage. Single multiple comparisons of data were done using one-way analysis of variance (one-way ANOVA) or Chi-squared tests as appropriate. Multiple association tests were done using a multivariable model with a binary logistic regression. In order to test multiple associations with ICU-mortality, age, SAPS 3, Sequential Organ Failure Assessment (SOFA), comorbidities, syndromic diagnosis, and lactate were used as independent covariates of the interest variable in four different models - each including acid-base variables as type of acid-base disorder, pH, SBE, or pCO_2_, which were individually evaluated. Hierarchical analyses were done, adding variables and comparing the area under the curve of the model and the Akaike information criterion of the two models. The higher the area under the curve and the lower the Akaike information criterion, the more appropriate the model.

A model for prediction of metabolic acidosis was done using binary logistic regression (comparing patients with SBE < - 2mEq/L with SBE ≥ 2mEq/L) and included prognostic covariables - namely, age, SAPS 3, total SOFA, Charlson score, syndromic diagnosis, maximum heart rate, maximum respiratory rate, maximum temperature, and need for MV. These variables were chosen because metabolic acidosis is associated with illness severity.^(18)^

We used bar-plots, chord-plots, alluvial-plots, and Sankey-plots^(22)^ to demonstrate occurrences, associations, and evolutionary data. Statistical analyses and graphs were performed using R software version 4.4.3.^(23)^ p values < 0.05 were considered significant.

A machine learning model to predict metabolic acidosis at the ICU-admission was built using a supervised (with a maximum of ten leaflets) logistic regression-based algorithm, with 80% and 20% of the sample used for the building and validation of the model, respectively. The same covariates cited above were used. The result was shown as a decision tree plot. The Scikit-learn version 1.6.1 module of Python version 3.13.2 was used to build the machine learning model.^(24)^

## RESULTS

### Epidemiological aspects at intensive care unit admission

During the study period, 3,046 patients were admitted to the ICU. The mean age was 52 ± 19 years, and 50% of patients were female. The main reason for ICU admission was circulatory shock (24%), followed by respiratory failure (17%). Table 1 shows the general baseline characteristics and requirements of vital organ support according to their acid-base diagnosis at ICU admission. The SAPS 3 score was higher in patients with respiratory and metabolic acidosis (61 ± 20), while participants with normal acid-base status had the lowest mean SAPS 3 score (53 ± 15).

**Table 1:** General characteristics, laboratory at admission, intensive care unit support, and outcomes of patients according to the acid-base diagnostics

		Whole group	Metabolic and respiratory acidosis	Metabolic and respiratory alkalosis	Metabolic acidosis	Metabolic acidosis and respiratory alkalosis	Metabolic alkalosis	Metabolic alkalosis and respiratory acidosis	Normal	Respiratory acidosis	Respiratory alkalosis	p value*
Sample		3,046	423	106	581	763	129	154	413	205	272	
General characteristics												
	Age (years)	52 ± 19	52 ± 19	50 ± 19	50 ± 19	52 ± 18	53 ± 20	55 ± 20	54 ± 18	55 ± 19	48 ± 19	< 0.001
	SAPS 3	57 ± 17	61 ± 20	55 ± 18	58 ± 17	60 ± 18	56 ± 17	55 ± 15	53 ± 15	54 ± 16	54 ± 15	< 0.001
	Total SOFA	7 ± 4	8 ± 4	7 ± 4	7 ± 4	7 ± 4	6 ± 4	6 ± 3	6 ± 3	6 ± 3	6 ± 4	< 0.001
	Females	1,523 (50)	220 (52)	54 (51)	271 (47)	395 (52)	75 (58)	85 (55)	198 (48)	104 (51)	121 (45)	0.098
	Weight (kg)	60.4 ± 5.5	60.9 ± 5.3	60.0 ± 3.8	60.1 ± 4.7	60.6 ± 5.8	61.5 ± 10.9	60.2 ± 4.3	60.0 ± 5.1	60.4 ± 5.5	60.3 ± 4.0	0.058
	Height (cm)	164.6 ± 10.3	165.4 ± 9.8	163.9 ± 8.9	165.1 ± 11.5	164.4 ± 9.7	163.7 ± 14.5	162.5 ± 10.1	164.9 ± 9.6	164.3 ± 9.8	164.6 ± 9.7	0.143
Acid-base and renal laboratory												
	pH admission	7.34 ± 0.12	7.19 ± 0.10	7.55 ± 0.08	7.30 ± 0.07	7.35 ± 0.10	7.45 ± 0.04	7.35 ± 0.09	7.40 ± 0.03	7.29 ± 0.07	7.47 ± 0.05	< 0.001
	pCO_2_ admission (mmHg)	39.9 ± 13.7	56.9 ± 12.4	26.9 ± 6.1	39.4 ± 2.8	28.1 ± 5.2	40.6 ± 2.6	60.8 ± 17.5	39.7 ± 2.9	55.5 ± 11.1	29.5 ± 5.6	< 0.001
	SBE admission (mEq/L)	- 3.6 ± 5.9	- 7.1 ± 4.5	4.4 ± 2.3	- 6.1 ± 3.7	- 8.2 ± 5.4	4.1 ± 2.1	6.0 ± 3.7	- 0.2 ± 1.1	- 0.2 ± 1.2	- 0.3 ± 1.1	< 0.001
	Lactate admission (mEq/L)	2.7 ± 2.4	3.2 ± 2.6	2.1 ± 1.2	2.9 ± 2.6	3.0 ± 3.0	2.0 ± 1.1	2.1 ± 1.4	2.1 ± 1.2	2.5 ± 1.9	2.1 ± 1.7	< 0.001
	Blood urea nitrogen (mg/dL)	39.5 ± 28.4	40.1 ± 25.8	26.1 ± 17.1	35.2 ± 29.2	44.8 ± 34.1	26.2 ± 20.1	27.2 ± 17.5	26.8 ± 24.9	26.9 ± 22.1	27.2 ± 22.3	< 0.001
	Creatinine (g/dL)	2.09 ± 2.47	2.08 ± 2.26	1.52 ± 1.55	2.46 ± 2.91	2.83 ± 3.08	1.44 ± 1.52	1.32 ± 1.63	1.49 ± 1.55	1.62 ± 1.81	1.51 ± 1.64	< 0.001
ICU support and outcomes												
	ICU-LOS (days)	9.2 ± 15.5	8.2 ± 8.9	9.3 ± 9.8	10.6 ± 28.1	7.8 ± 8.2	10.4 ± 13.1	13.8 ± 19.8	9.2 ± 11.4	8.8 ± 10.2	8.4 ± 8.1	0.001
	Vasopressors admission	722 (24)	138 (33)	11 (10)	162 (28)	190 (25)	21 (16)	22 (14)	86 (21)	46 (22)	46 (17)	< 0.001
	Vasopressors any moment	1,409 (47)	220 (52)	39 (37)	300 (52)	373 (49)	57 (44)	60 (39)	160 (39)	89 (43)	111 (41)	< 0.001
	MV admission	671 (22)	136 (32)	18 (17)	148 (26)	136 (18)	27 (21)	40 (26)	83 (20)	45 (22)	38 (14)	< 0.001
	MV any moment	1,231 (41)	214 (51)	36 (34)	275 (47)	263 (35)	50 (39)	78 (51)	150 (36)	80 (39)	85 (31)	< 0.001
	Sedatives admission	298 (10)	63 (15)	6 (6)	80 (14)	67 (9)	9 (7)	12 (8)	33 (8)	15 (7)	13 (5)	< 0.001
	Sedatives any moment	693 (23)	130 (31)	17 (16)	151 (26)	167 (22)	31 (24)	37 (24)	78 (19)	42 (21)	40 (15)	< 0.001
	RPT admission	236 (8)	39 (9)	6 (6)	55 (10)	88 (12)	6 (5)	3 (2)	17 (4)	10 (5)	12 (5)	< 0.001
	RPT any moment	611 (20)	87 (21)	16 (15)	134 (23)	206 (27)	16 (12)	26 (17)	57 (14)	28 (14)	41 (15)	< 0.001
	Exclusive palliative care	354 (12)	55 (13)	7 (7)	75 (13)	100 (13)	17 (13)	17 (11)	34 (8)	24 (12)	25 (9)	0.130
	ICU death	651 (21)	108 (26)	11 (10)	132 (23)	209 (27)	27 (21)	20 (13)	68 (17)	33 (16)	43 (16)	< 0.001
Main causes of ICU admission												
	Shock syndrome	730 (24)	120 (28)	18 (17)	160 (28)	219 (29)	24 (19)	32 (21)	67 (16)	44 (22)	46 (17)	< 0.001
	Respiratory failure	523 (17)	88 (21)	14 (13)	87 (15)	160 (21)	17 (13)	31 (20)	60 (15)	30 (15)	36 (13)	0.003
	High postoperative risk	461 (15)	73 (17)	14 (13)	90 (16)	77 (10)	19 (15)	18 (12)	82 (20)	48 (23)	40 (15)	< 0.001
	Sepsis	393 (13)	39 (9)	12 (12)	68 (12)	129 (17)	19 (15)	21 (15)	46 (11)	23 (11)	36 (13.2)	0.014
	Acute neurological disorders	177 (6)	20 (5)	5 (6)	25 (4)	45 (6)	10 (8)	9 (6)	31 (8)	18 (9)	14 (5)	0.255
	Others†	1,135 (37)	150 (36)	52 (49)	216 (37)	245 (32)	55 (43)	62 (40)	165 (40)	67 (33)	123 (45)	< 0.001
Comorbidities												
	Arterial hypertension	565 (19)	85 (20)	16 (15)	108 (19)	129 (17)	22 (17)	29 (19)	90 (22)	44 (22)	42 (15)	0.357
	Diabetes mellitus	356 (12)	53 (13)	12 (11)	70 (12)	85 (11)	15 (12)	21 (14)	43 (10)	30 (15)	27 (10)	0.823
	AIDS	240 (8)	35 (8)	11 (10)	40 (7)	57 (8)	8 (6)	13 (8)	40 (10)	19 (9)	17 (6)	0.655
	Cirrhosis	218 (7)	33 (8)	9 (9)	38 (7)	48 (6)	8 (6)	12 (8)	34 (8)	17 (8)	19 (7)	0.925
	Metastatic neoplasms	226 (7)	35 (8)	5 (5)	33 (6)	55 (7)	10 (8)	12 (8)	41 (10)	18 (9)	17 (6)	0.333
	Heart failure	180 (6)	30 (7)	7 (7)	36 (6)	41 (5)	3 (2)	16 (10)	25 (6)	12 (6)	10 (4)	0.125
	COPD	196 (6)	33 (8)	8 (8)	33 (6)	39 (5)	7 (5)	13 (8)	29 (7)	15 (7)	19 (7)	0.626
	Atrial fibrillation	179 (6)	26 (6)	6 (6)	31 (5)	40 (5)	6 (5)	11 (7)	29 (7)	16 (8)	14 (5)	0.825
	Onco-hematological diseases	114 (4)	18 (4)	2 (2)	18 (3)	31 (4)	3 (2)	7 (5)	17 (4)	8 (4)	10 (4)	0.908

SAPS - Simplified Acute Physiological Score; SOFA - Sequential Organ Failure Assessment; pCO_2_ - partial pressure of carbon dioxide; SBE - standard base excess; ICU - intensive care unit; LOS - length-of-stay; MV - mechanical ventilation; RPT - renal replacement therapy; AIDS - Acquired Immunodeficiency Syndrome; COPD - chronic obstructive pulmonary disease.

* p value of analysis of variance one-way or Chi-squared test as appropriate among the group of acid-base diagnostics;

† others are a group of syndromes: polytrauma, cardiogenic shock, severe electrolyte derangements, aortic pathologies, severe dermatologic diseases, burns, digestive hemorrhage, acute and acute on chronic liver failure, severe pancreatitis, diabetic ketoacidosis, severe intoxications, and thyroid storm. Results expressed as n, mean ± standard deviation or n (%).

**Figure 1:**
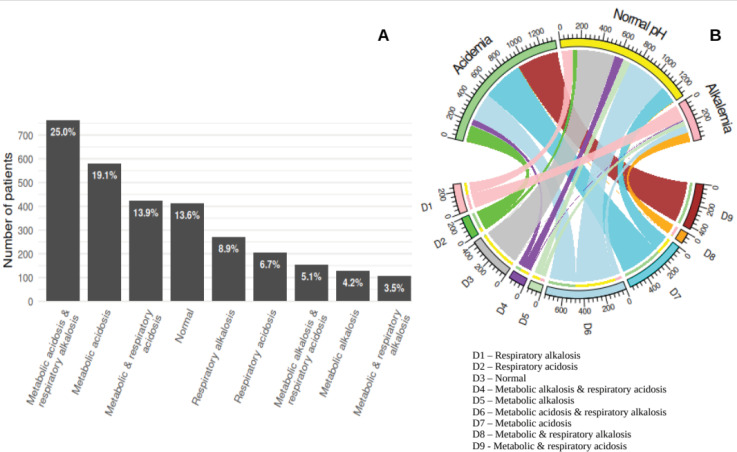
Distribution of acid-base diagnostics at intensive care unit admission.

Figure 1A presents the incidence of each acid-base diagnosis at ICU admission. Metabolic acidosis, either pure or combined with other disturbances, was the most frequent derangement (58.0%), followed by respiratory alkalosis (37.6%), respiratory acidosis (25.7%), and metabolic alkalosis (12.8%). Only 13.6% of subjects had normal acid-base status. Metabolic acidosis with respiratory alkalosis was the most frequent combined acid-base diagnosis, occurring in 25% of patients.

(A) The bar plot shows the metabolic and respiratory diagnostics; (B) the chord plot shows the interaction of the metabolic and respiratory diagnostics with the pH.

The numbers around the chord plot are the patients in each acid-base diagnostic category. The percentages in panel A denote the representation of the analyzed acid-base diagnostic over the 3,046 patients. Acidemia denotes a pH < 7.35; alkalemia denotes a pH > 7.45; and normal pH denotes a 7.35 ≤ pH ≤ 7.45. Metabolic acidosis denotes a standard base excess < - 2mEq/L; metabolic alkalosis denotes a standard base excess > 2mEq/L; respiratory acidosis denotes a partial pressure of carbon dioxide > 45mmHg; respiratory alkalosis denotes a partial pressure of carbon dioxide < 35mmHg and normal denotes a -2mEq/L ≤ standard base excess ≤ 2mEq/L and 35mmHg ≤ partial pressure of carbon dioxide ≤ 45mmHg. Statistical analyses and graphs were performed using R software version 4.4.3

Figure 1B shows the relationship between acid-base diagnoses and pH categories (normal, alkalemia, and acidemia). Of note, most patients with metabolic acidosis combined with respiratory alkalosis had normal pH values.

### Acid-base characteristics at admission and the association with intensive care unit mortality

Table 2 shows the multivariable regression models evaluating risk factors for ICU mortality. Model 1 included clinical characteristics of ICU admission (age, SAPS 3, total SOFA, comorbidities, and syndromic diagnosis) and laboratory data (lactate). In addition to these factors, the remaining models included the combined respiratory and metabolic diagnosis (Model 2); the pH (Model 3); the SBE (Model 4); or the pCO_2_ (Model 5). Age, SAPS3, SOFA, and lactate were independently associated with ICU mortality in all models. Interestingly, there was no association between pH (Model 3) and pCO_2_ (Model 5) and mortality. The only combined acid-base diagnosis independently associated with mortality was metabolic alkalosis combined with respiratory alkalosis (odds ratio [OR] 0.621, 95% confidence interval [95%CI] 0.265 - 1.000; p = 0.044), with a protective effect (Model 2). Higher SBE was also significantly associated with lower mortality in Model 4 (OR 0.973, 95%CI: 0.956 - 0.990; p < 0.001).

**Table 2:** Association with intensive care unit death of five models with different acid-base scenarios, with admission data

		Model 1		Model 2		Model 3		Model 4		Model 5	
		OR/AUC (95%CI)	p value	OR/AUC (95%CI)	p value	OR/AUC (95%CI)	p value	OR/AUC (95%CI)	p value	OR/AUC (95%CI)	p value
Age		1.016 (1.011 - 1.022)	< 0.001	1.016 (1.011 - 1.022)	< 0.001	1.016 (1.011 - 1.022)	< 0.001	1.017 (1.011 - 1.023)	< 0.001	1.017 (1.011 - 1.023)	< 0.001
SAPS 3		1.044 (1.038 - 1.051)	< 0.001	1.043 (1.037 - 1.050)	< 0.001	1.044 (1.038 - 1.051)	< 0.001	1.042 (1.035 - 1.048)	< 0.001	1.042 (1.035 - 1.048)	< 0.001
Total SOFA		1.169 (1.140 - 1.199)	< 0.001	1.164 (1.134 - 1.194)	< 0.001	1.169 (1.140 - 1.199)	< 0.001	1.159 (1.130 - 1.189)	< 0.001	1.159 (1.130 - 1.189)	< 0.001
Modified Charlson		0.953 (0.901 - 1.008)	0.124	0.960 (0.906 - 1.015)	0.159	0.953 (0.901 - 1.008)	0.116	0.957 (0.905 - 1.012)	0.123	0.957 (0.905 - 1.012)	0.135
Syndromic diagnosis		0.980 (0.929 - 1.034)	0.646	0.991 (0.939 - 1.047)	0.756	0.980 (0.929 - 1.034)	0.689	0.995 (0.943 - 1.050)	0.857	0.995 (0.943 - 1.050)	0.674
pH		----------		----------		0.621 (0.265 - 1.456)	0.273	----------		----------	
pCO_2_		----------		----------		----------		----------		0.973 (0.956 - 0.990)	0.227
SBE		----------		----------		----------		0.973 (0.956 - 0.990)	< 0.001	----------	
Lactate		1.010 (1.005 - 1.014)	< 0.001	1.009 (1.004 - 1.013)	< 0.001	1.010 (1.005 - 1.014)	< 0.001	1.007 (1.003 - 1.012)	< 0.001	1.007 (1.003 - 1.012)	< 0.001
Acid-base diagnoses											
	Metabolic and respiratory alkalosis	----------		0.427 (0.194 - 0.869)	0.025	----------		----------		----------	
	Metabolic acidosis	----------		1.059 (0.733 - 1.537)	0.761	----------		----------		----------	
	Metabolic acidosis and respiratory alkalosis	----------		1.225 (0.868 - 1.742)	0.253	----------		----------		----------	
	Metabolic alkalosis	----------		1.157 (0.652 - 2.009)	0.611	----------		----------		----------	
	Metabolic alkalosis and respiratory acidosis	----------		0.683 (0.374 - 1.205)	0.201	----------		----------		----------	
	Metabolic and respiratory acidosis	----------		0.908 (0.613 - 1.348)	0.630	----------		----------		----------	
	Respiratory acidosis	----------		0.890 (0.539 - 1.448)	0.643	----------		----------		----------	
	Respiratory alkalosis	----------		0.972 (0.610 - 1.535)	0.903	----------		----------		----------	
AUC of the model		0.810 (0.791 - 0.829)	< 0.001	0.812 (0.794 - 0.831)	< 0.001	0.810 (0.791 - 0.828)	< 0.001	0.811 (0.792 - 0.829)	< 0.001	0.811 (0.792 - 0.829)	< 0.001

OR - odds ratio; AUC - area under the curve; 95%CI - 95% confidence interval; SAPS - Simplified Acute Physiological Score; SOFA - Sequential Organ Failure Assessment; pCO_2_ - partial pressure of carbon dioxide; SBE - standard base excess.

Model 1 - clinical main characteristics associated with death plus lactate; Model 2 - Model 1 plus intensive care unit admission acid-base diagnosis; Model 3 - Model 1 plus pH; Model 4 - Model 1 plus standard base excess; and Model 5 - Model 1 plus pCO_2_. All models were built using a binary logistic regression.

To explore the characteristics of patients with metabolic acidosis at ICU admission, table 1S (Supplementary Material) compares clinical and laboratory characteristics and use of organ support of patients admitted with and without metabolic acidosis. Although the patients presenting with metabolic acidosis were younger, they were more severely ill, needed more organ support, and had higher ICU mortality compared to patients without metabolic acidosis.

### Epidemiology of the evolution of acid-base derangements

**Figure 2:**
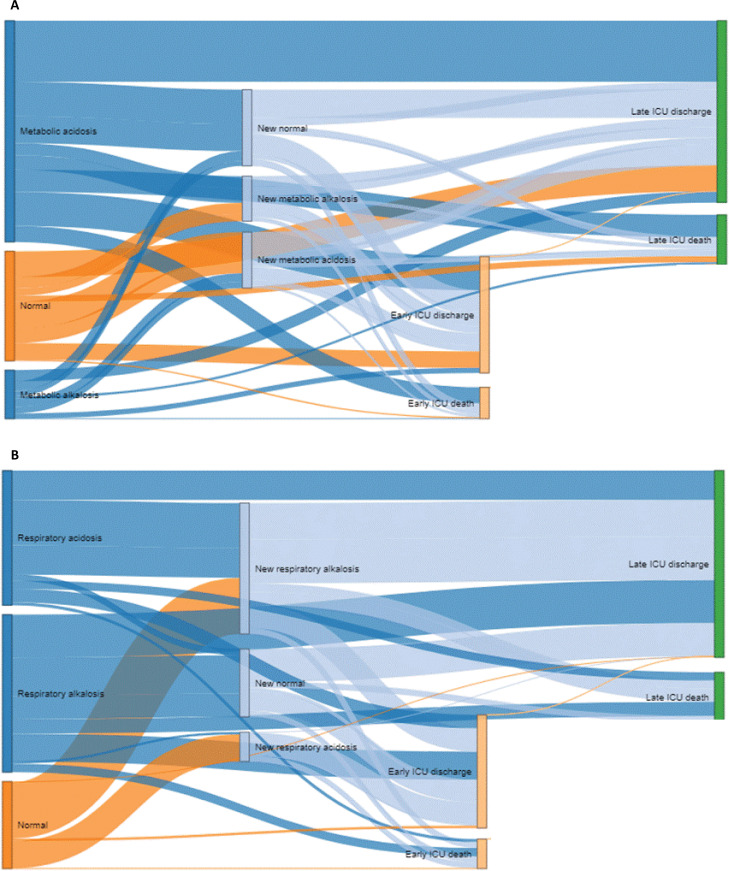
Sankey plot showing the metabolic (A) and respiratory (B) components evolution of the acid-base metabolism during the intensive care unit stay of all patients until intensive care unit discharge or death.

A Sankey plot illustrates the evolutive flow of patients through the ICU stay according to the metabolic (Figure 2A) or respiratory diagnosis (Figure 2B). It demonstrates the proportion of patients in each category of admission acid-base status and their trajectories: maintenance of admission diagnosis, development of a new disorder (acidosis or alkalosis), or normalization. Possible outcomes (early or late discharge or death) are shown for each pathway. The main observation is that most patients developed a new acid-base diagnosis during ICU stay. Also, neither early nor late outcomes appear to be clearly related to a specific trajectory of evolution of the acid-base status.

ICU - intensive care unit.

Early death or discharge was considered up to the fourth intensive care unit day. Late death or discharge was considered if it happened with an intensive care unit stay of 5 or more days. The acid-base disturbances were considered as follows: metabolic component => acidosis if standard base excess < - 2mEq/L; normal if - 2mEq/L ≤ standard base excess ≤ 2mEq/L; and alkalosis if standard base excess > 2mEq/L. Respiratory component => acidosis if partial pressure of carbon dioxide > 45mmHg; normal if 35mmHg ≤ partial pressure of carbon dioxide ≤ 45mmHg; and alkalosis if partial pressure of carbon dioxide < 35mmHg. Statistical analyses and graphs were performed using R software version 4.4.3

As the metabolic component at ICU admission (SBE) was independently associated with mortality, its evolutive behavior was explored in patients with early outcome (< 5 days of ICU-stay) in figure 1S (Supplementary Material); and in patients with late outcome (≥ 5 days of ICU-stay) in figure 2S (Supplementary Material). Almost half of the patients who died, either early or late, were admitted with metabolic acidosis. Furthermore, to get more granularity with SBE evolution, we arbitrarily chose 5 days to illustrate the changes in the acid-base metabolic component during ICU stay and the outcome for each trajectory through an alluvial plot (Figure 3AS and 3BS - Supplementary Material). For each arbitrary category of admission SBE, the change in metabolic acid-base status is shown throughout ICU stay, as well as the outcome for each pathway. The main observations were that the variation in acid-base status was more prominent on the first day of ICU stay and that there was a slight increase in metabolic alkalosis in the overall population. There was no clear visual association of the metabolic temporal behavior with the outcome.

Finally, figure 4S (Supplementary Material) shows the acid-base temporal changes during ICU-stay, according to pH, SBE, and pCO_2_ evolution. It is important to note that 84% of patients admitted with metabolic acidosis improved during the stay, and 31% of patients without metabolic acidosis at ICU-admission presented any degree of metabolic acidosis during the stay. Most (82%) patients with respiratory acidosis lowered pCO_2_ after 4 days. In contrast, a minority (19%) of patients admitted with respiratory alkalosis had increased pCO_2_. Overall, the incidence of acidemia and metabolic acidosis decreased from the first to the last measurement during the ICU stay.

### Evolutive acid-base derangements associated with intensive care unit mortality

We evaluated the association between the behavior of the respiratory component of the acid-base status and ICU mortality. Table 2S (Supplementary Material) shows that there was no significant association between maximum pCO_2_ variation and mortality. The metabolic temporal behavior during ICU stay is shown in table 3. Standard base excess maximum variation from admission to any point of ICU stay up to the fourth ICU day was not associated with mortality when analyzed with admission clinical and laboratory mortality predictors, including SBE at ICU admission. By contrast, SBE maximum variation slightly adds prediction power to classical clinical and laboratory ICU mortality predictors (including the SBE) at admission when analyzed in patients who stayed 5 or more days in the ICU. In the last observation, improvement of SBE was associated with reduced ICU mortality.

**Table 3:** Analysis of survival association of intensive care unit-admission standard base excess of patients categorized according to the intensive care unit-length-of-stay, and hierarchical survival association analysis of adding maximum standard base excess variation through the first 5 days in intensive care unit

	< 5 days of ICU-LOS (n = 1,198 patients)				≥ 5 days of ICU-LOS (n = 1,848 patients)			
	Model 1 - without SBE maximum variation		Model 2 - with SBE maximum variation		Model 3 - without SBE maximum variation		Model 4 - with SBE maximum variation	
	OR/AUC (95%CI)	p value	OR/AUC (95%CI)	p value	OR/AUC (95%CI)	p value	OR/AUC (95%CI)	p value
Age	1.022 (1.011 - 1.033)	< 0.001	1.021 (1.010 - 1.032)	< 0.001	1.016 (1.009 - 1.024)	< 0.001	1.016 (1.009 - 1.023)	< 0.001
SAPS 3	1.065 (1.053 - 1.078)	< 0.001	1.065 (1.053 - 1.078)	< 0.001	1.030 (1.022 - 1.038)	< 0.001	1.029 (1.021 - 1.037)	< 0.001
Total SOFA	1.191 (1.143 - 1.242)	< 0.001	1.191 (1.143 - 1.242)	< 0.001	1.140 (1.101 - 1.179)	< 0.001	1.138 (1.099 - 1.177)	< 0.001
Modified Charlson	0.863 (0.770 - 0.967)	0.011	0.862 (0.769 - 0.967)	0.011	1.002 (0.939 - 1.070)	0.950	1.002 (0.938 - 1.069)	0.963
Syndromic diagnosis	0.993 (0.896 - 1.100)	0.887	0.995 (0.898 - 1.103)	0.928	0.992 (0.930 - 1.057)	0.798	0.991 (0.929 - 1.057)	0.781
SBE	0.937 (0.905 - 0.969)	< 0.001	0.924 (0.886 - 0.963)	< 0.001	0.989 (0.968 - 1.010)	0.313	0.977 (0.953 - 1.001)	0.063
Lactate	1.014 (1.005 - 1.022)	0.001	1.014 (1.006 - 1.022)	0.001	1.003 (0.996 - 1.009)	0.408	1.003 (0.997 - 1.010)	0.294
Maximum SBE variation	-----------	-------	0.973 (0.928 - 1.020)	0.250	-----------	-------	0.964 (0.930 - 1.000)	0.050
AUC of the model	0.903 (0.881 - 0.924)	< 0.001	0.903 (0.881 - 0.925)	< 0.001	0.744 (0.717 - 0.771)	< 0.001	0.746 (0.719 - 0.773)	< 0.001

ICU - intensive care unit; LOS - length-of-stay; SBE - standard base excess; OR - odds ratio; AUC - area under the curve; 95%CI - 95% confidence interval; SAPS - Simplified Acute Physiological Score; SOFA - Sequential Organ Failure Assessment. The maximum standard base excess variation was calculated as the maximum positive difference between standard base excess from day 1 to 5 (or up to the last ICU-day) subtracted from the standard base excess of the patient's intensive care unit admission. The maximum standard base excess variation of patients with intensive care unit -length-of-stay < 5 days was: median = 1.1mEq/L, minimum = -5.1mEq/L, and maximum = 34.9mEq/L. The maximum standard base excess variation of patients with intensive care unit -length-of-stay ≥ 5 days was: median = 0.4mEq/L, minimum = 0mEq/L, and maximum = 34.6mEq/L. All models were built using a binary logistic regression. The hierarchical analysis of adding maximum standard base excess variation to models 1 and 2 resulted in the following: the Akaike information criteria of model 1 was 740, and the Akaike information criteria of model 2 was 740. The comparative analysis of variance of models 1 and 2 resulted in a p value = 0.250. The Akaike information criteria of model 3 was 1692, the Akaike information criteria of model 4 was 1690. The comparative analysis of variance of models 3 and 4 resulted in a p value = 0.010.

### Prediction of metabolic acidosis diagnosis at intensive care unit admission and during the intensive care unit stay

Higher SBE at ICU admission was associated with reduced mortality; therefore, we sought clinical and laboratory factors to predict the occurrence of metabolic acidosis at admission. Furthermore, despite the lack of a mortality association, we also explored the factors that predict new metabolic acidosis diagnosis during ICU stay. Table 3S (Supplementary Material) shows an association between the presence of metabolic acidosis at ICU admission with lower age, higher disease severity, more organ dysfunctions, the presence of circulatory shock, higher creatinine, higher heart rate, lower maximum body temperature, and lower respiratory rate. A new metabolic acidosis diagnosis after ICU admission was associated with lower admission SBE, more organ dysfunction at ICU admission, and the need for MV.

A decision tree (Figure 5S - Supplementary Material) was built to explore the complexity of the association between metabolic acidosis predictors and metabolic acidosis diagnosis at ICU admission. The model had a moderate mean accuracy of 0.62. The primary split reflected renal function - patients with a creatinine value > 4.055mg/dL had a higher chance of presenting metabolic acidosis at ICU admission. Moreover, secondary splits occurred with organ failure, age, and temperature. Patients with creatinine ≤ 4.055mg/dL and total SOFA > 10.5 also had a high chance of having the diagnosis of metabolic acidosis at ICU admission. Among patients with creatinine ≤ 2.015 and total SOFA ≤ 10.5, age > 49.5 years increased the risk of metabolic acidosis. Lower temperatures (≤ 36.75) increased the risk in younger patients with less organ dysfunction.

## DISCUSSION

Among the 3,046 critically ill patients of this dataset, 58% had metabolic acidosis as a single diagnosis or associated with another acid-base diagnosis at ICU admission. The combination of metabolic and respiratory alkalosis was associated with reduced mortality. The higher SBE at ICU admission was associated with lower ICU mortality. The metabolic acidosis occurrence was followed by respiratory alkalosis and respiratory acidosis, respectively in 37.6% and 25.7% of patients. After ICU admission, 31% of patients developed a new metabolic acidosis, and 59% of them developed a new respiratory acidosis. At admission, 13% of patients in this cohort had metabolic alkalosis, and 44% of the patients who were admitted without metabolic alkalosis developed it during ICU stay. Lower age, higher disease severity, more organ dysfunctions, the presence of circulatory shock, higher creatinine, higher heart rate, lower maximum body temperature and lower respiratory rate were associated with metabolic acidosis at ICU admission

The current literature on the epidemiology of acid-base derangements in critically ill patients is somewhat contradictory, with findings pointing in opposite directions. Norwegian ICUs’ data^(11)^ has shown metabolic alkalosis as the most common acid-base disturbance, meanwhile South-African^(9)^ and Indian^(10)^ ICUs reported metabolic acidosis as the most common one. However, all of them were consistent with our findings. The South-African admission incidence of metabolic acidosis diagnosis was 64.5%,^(9)^ similar to the 58% in our cohort. Moreover, 44% of our patients without metabolic alkalosis at admission developed this acid-base derangement, like Norwegian's^(11)^ findings, which reported metabolic alkalosis as frequent during the ICU stay. This metabolic alkalosis probably occurred because of a progressive rise of SBE due to the sodium elevation and chloride reduction, attributed to the use of loop diuretics.^(11)^

Previous studies support the association of metabolic acidosis at ICU admission with a higher mortality.^(3,4,6,25)^ We expected that the rise of SBE during the ICU stay should be related to improved survival; however, this association was only found in patients who stayed 5 or more days in the ICU. This finding could be explained by the higher length-of-stay of patients with isolated metabolic acidosis (Table 1), who were more severely ill patients, and were expected to stay longer in the ICU; otherwise, extremely ill patients who died during the first four days of ICU stay may not have had time to recover the SBE. This finding goes against the Australian persistent critical illness definition,^(26)^ where there is a time point at which acute illness severity loses the capacity to predict outcomes. However, we are analyzing only one dimension of the complex issue of ICU outcome prediction.

Additionally, we found a frequent diagnosis of a new metabolic alkalosis during the ICU stay (44%), which was associated in an evolutive view with improved outcomes. This contrasts with the current literature, in which the diagnosis of metabolic alkalosis is associated with a higher mortality.^(27,28)^ We ascribe this finding to metabolic alkalosis as secondary to the improvement of the SBE.

Another interesting finding of the present study was the clinical and laboratory predictors of metabolic acidosis at ICU admission. These predictors were also disease severity markers, showing that the presence of metabolic acidosis is associated with organ dysfunction severity^(6,18)^ and endogenous inflammatory pathways activation, which is an additional contributor to the disease severity.^(29)^

Our study has several limitations: the observational design precludes causal inference, in this way, our results are only for monitorization and must not be a background for clinical interventions; data were collected solely from ICU patients and, thus, admission to the ICU may act as a collider in the association of acid-base disturbances with mortality, because patients with an abnormal blood gas analysis are more likely to be admitted to the ICU; we did not calculate practical strong ion difference and substantial ion gap; data about in-hospital or long term outcomes are lacking; and we do not provide data about acid-base compensation; however, the presented data are bedside practical tools for daily management of critically ill patients.

## CONCLUSION

Metabolic acidosis alone or combined with other acid-base derangements was frequent at the time of intensive care unit admission. Lower standard base excess at intensive care unit admission was associated with higher mortality; moreover, standard base excess recovery during intensive care unit stay is associated with reduced mortality in patients who stayed 5 or more days in the intensive care unit. Clinical and laboratory data associated with higher disease severity predicted the presence of metabolic acidosis at the time of intensive care unit admission and stay.

## Data Availability

The contents will be made available at the time of publication of the article. Database: https://drive.google.com/file/d/1y-oAgD-KsHK_8hMzEnNPkbwoXNanXy9W/view?usp=sharing
