# Supplementary material for: Evolutive acid-base derangements in critically ill patients: epidemiological aspects, association with mortality and metabolic acidosis prediction
Source: Crit Care Sci. 2026 Jan 9;38:e20260160. doi: 10.62675/2965-2774.20260160 (PMC12977211; doi:10.62675/2965-2774.20260160)
Supplement: Supplementary file 1 [file 2965-2774-ccsci-38-e20260160-suppl1.pdf]

https://doi.org/10.62675/2965-2774.20260160

SUPPLEMENTARY MATERIAL

Evolutive acid-base derangements in critically ill patients: epidemiological aspects, association with mortality and metabolic acidosis prediction

Carine Carrijo de Faria[1], Caterina Lure Nema Paiva[1], Luiz Marcelo Almeida de Araujo[1], Luis Carlos Maia Cardozo Júnior[1], Marcelo Park[1]

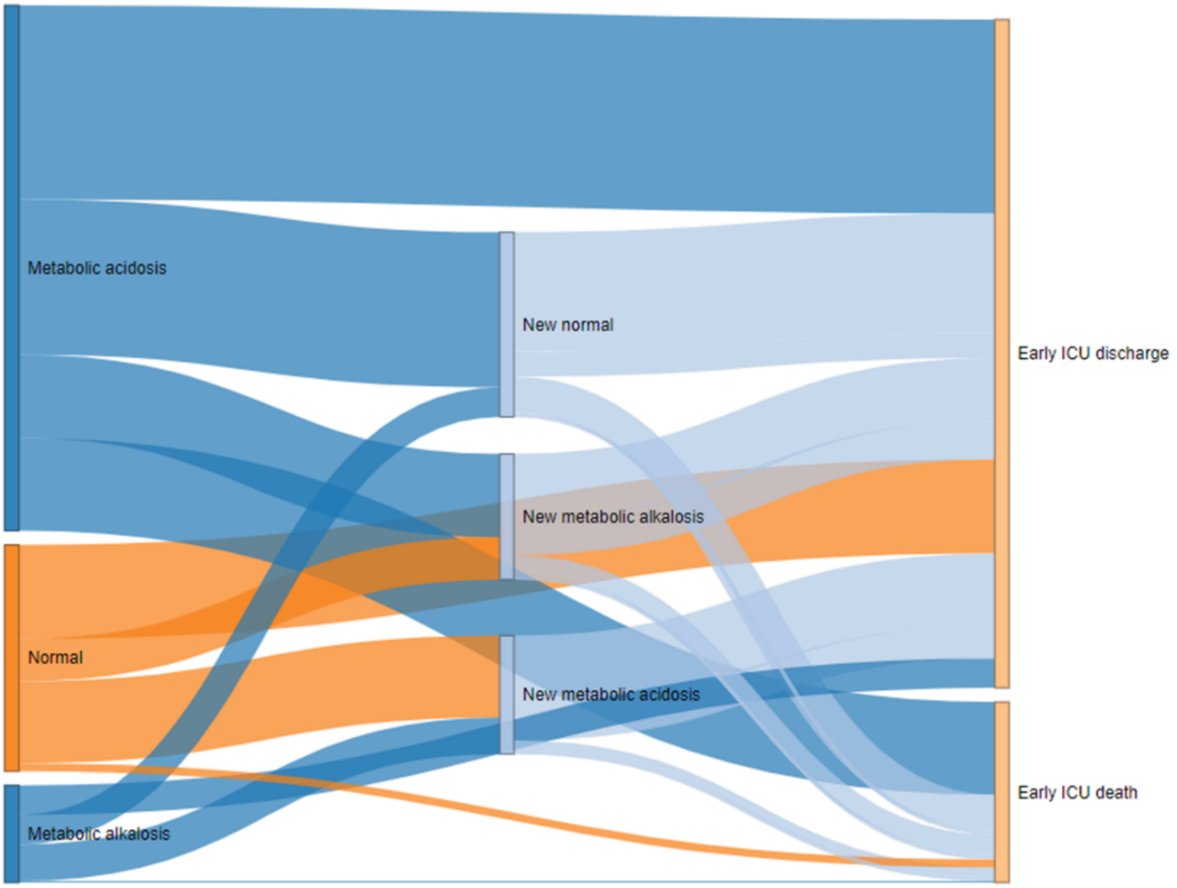

ICU - intensive care unit.

Figure 1S - Sankey plot showing the metabolic component evolution of the acid-base metabolism during the intensive care unit stay of patients who stayed lesser than five days in intensive care unit, configuring our definition of early outcome.

The acid-base disturbances were considered as follows: metabolic component => acidosis if SBE < - 2mEq/L; normal if - 2mEq/L ≤ standard base excess ≤ 2mEq/L; and alkalosis if standard base excess > 2mEq/L. Statistical analyses and graphs were performed using R software version 4.4.3

This is an open access article under the CC BY license (https://creativecommons.org/licenses/by/4.0/).

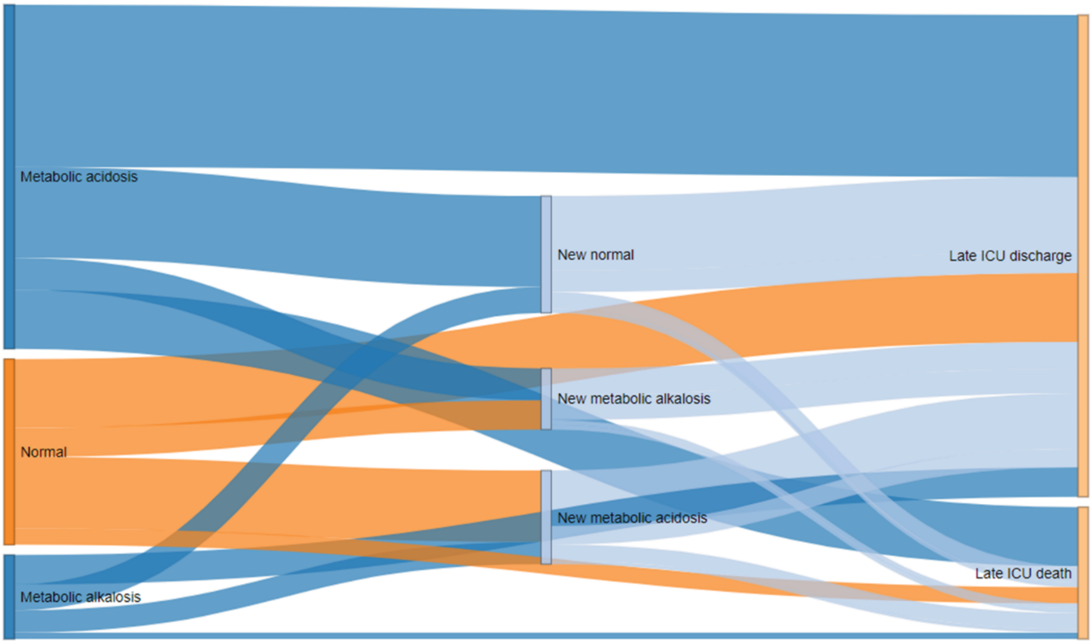

ICU - intensive care unit.

Figure 2S - Sankey plot showing the metabolic component evolution of the acid-base metabolism during the intensive care unit stay of patients who stayed five days or more in intensive care unit, configuring our definition of late outcome.

The acid-base disturbances were considered as follows: metabolic component => acidosis if standard base excess < - 2mEq/L; normal if - 2mEq/L ≤ standard base excess ≤ 2mEq/L; and alkalosis if standard base excess > 2mEq/L. Statistical analyses and graphs were performed using R software version 4.4.3

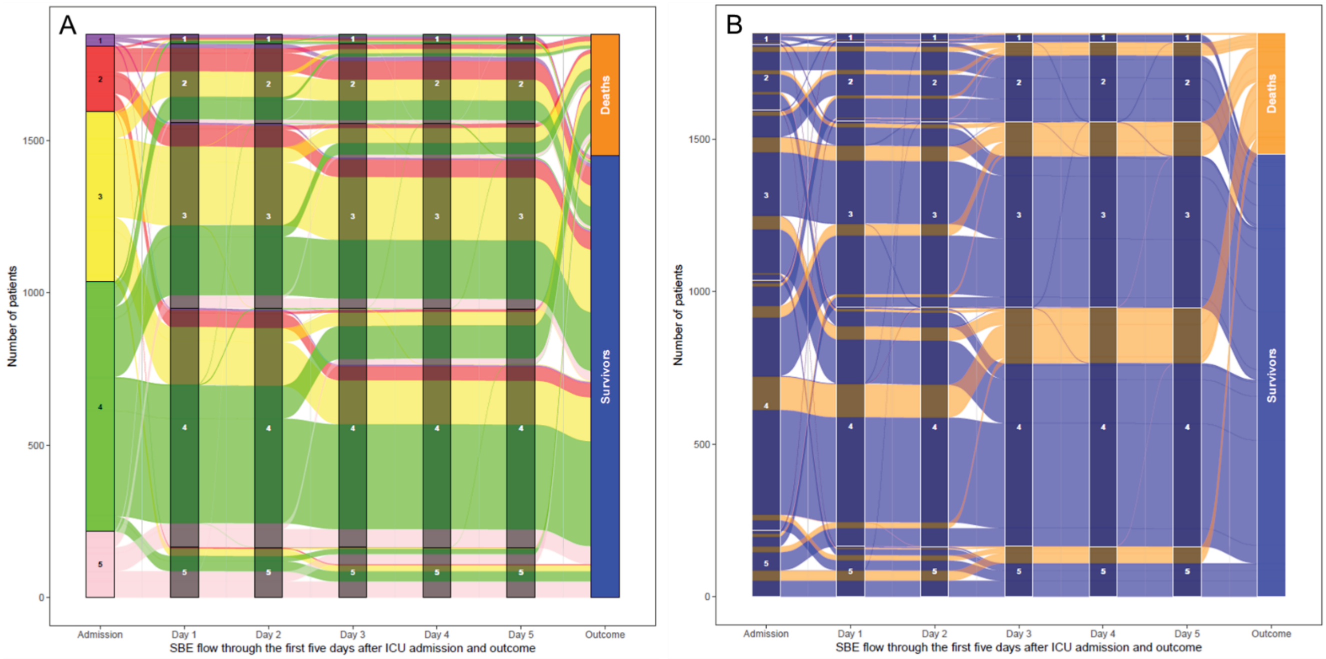

1) SBE > 8 mEq/L
2) 2 mEq/L < SBE ≤ 8 mEq/L
3) - 2 mEq/L ≤ SBE ≤ 2 mEq/L
4) - 10 mEq/L < SBE < - 2 mEq/L
5) SBE ≤ - 10 mEq/L

SBE - standard base excess; ICU - intensive care unit.

Figure 3S - Alluvial plot showing the standard base excess flow through the first 5 days after the intensive care unit admission.

(A) Standard base excess flow highlights the standard base excess range at intensive care unit admission; (B) The same standard base excess flow highlights the survival of the intensive care unit. The sample size analyzed in this figure was 1,848 patients who stayed at least 5 days in the intensive care unit. Statistical analyses and graphs were performed using R software version 4.4.3

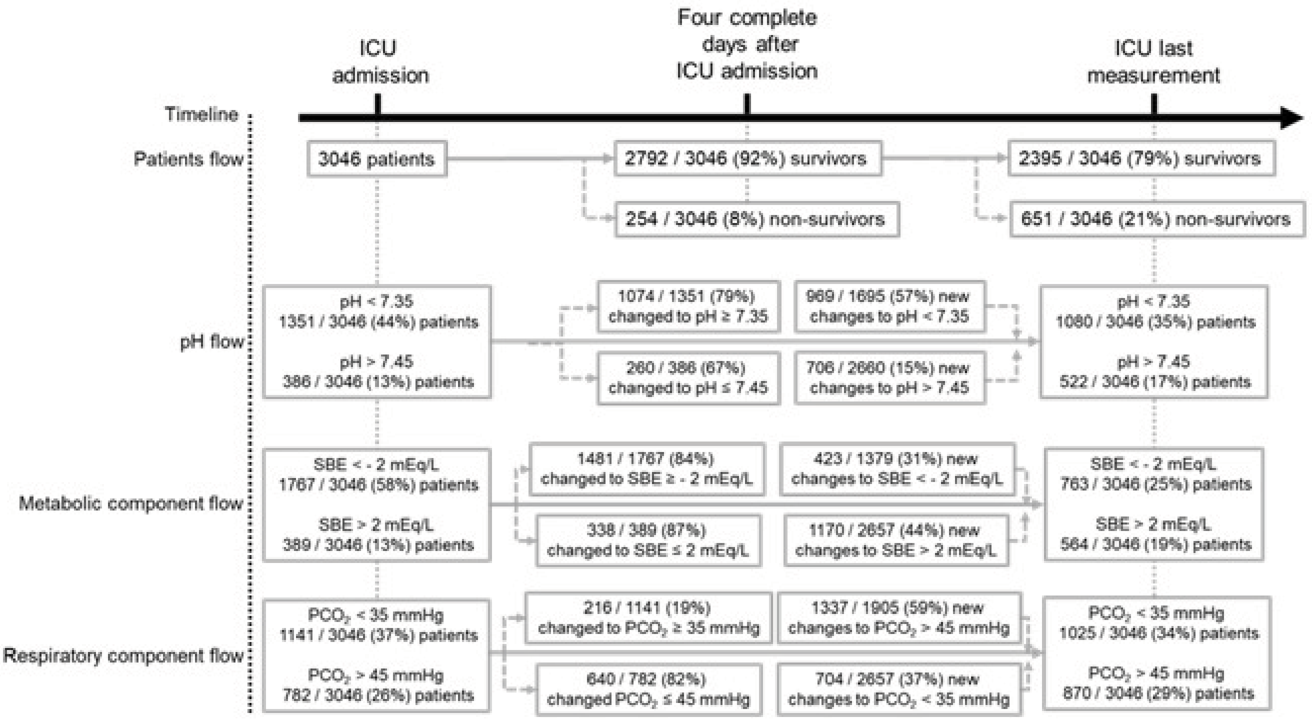

ICU - intensive care unit; SBE - standard base excess; pCO_2 - partial pressure of carbon dioxide.

Figure 4S - Flowchart showing the evolution of patients, pH, metabolic and respiratory changes during the intensive care unit stay.

New changes denote alterations of pH, standard base excess, or pCO_2 that were not present at intensive care unit admission and occurred after intensive care unit admission.

Table 1S - General characteristics, laboratory at admission, intensive care unit support and outcomes of patients according to the presence of metabolic acidosis

	Whole group	SBE ≥ - 2 mEq/L	SBE < - 2 mEq/L	p value*
Sample	3,046	1,279	1,767	
General characteristics				
Age (years)	52 ± 19	52.58 ± 19.09	50.92 ± 18.57	0.016
SAPS 3	57 ± 17	53.98 ± 15.60	59.59 ± 18.35	< 0.001
Total SOFA	7 ± 4	6.15 ± 3.52	7.07 ± 4.01	< 0.001
Females	1,523 (50)	637 (49.8)	886 (50.1)	0.883
Weight (kg)	60.4 ± 5.5	60.28 ± 5.67	60.49 ± 5.35	0.301
Height (cm)	164.6 ± 10.3	164.22 ± 10.23	164.83 ± 10.36	0.107
Acid-base and renal laboratory				
pH admission	7.34 ± 0.12	7.41 ± 0.09	7.30 ± 0.12	< 0.001
pCO_2 admission (mmHg)	39.9 ± 13.7	41.61 ± 13.97	38.69 ± 13.37	< 0.001
SBE admission (mEq/L)	- 3.6 ± 5.9	1.36 ± 3.04	-7.22 ± 4.76	< 0.001
Lactate admission (mEq/L)	2.7 ± 2.4	2.15 ± 1.45	3.00 ± 2.79	< 0.001
Blood urea nitrogen (mg/dL)	39.5 ± 28.4	53.69 ± 40.24	76.29 ± 64.03	< 0.001
Creatinine (g/dL)	2.09 ± 2.47	1.49 ± 1.62	2.53 ± 2.86	< 0.001
ICU support and outcomes				
ICU-LOS (days)	9.2 ± 15.5	9.63 ± 12.12	8.83 ± 17.56	0.162

Continue...

...continuation

Vasopressors admission	722 (24)	232 (18.1)	490 (27.7)	< 0.001
Vasopressors any moment	1,409 (47)	516 (40.3)	893 (50.5)	< 0.001
MV admission	671 (22)	251 (19.6)	420 (23.8)	0.007
MV any moment	1,231 (41)	479 (37.5)	752 (42.6)	0.005
Sedatives admission	298 (10)	88 (6.9)	210 (11.9)	< 0.001
Sedatives any moment	693 (23)	245 (19.2)	448 (25.4)	< 0.001
RRT admission	236 (8)	54 (4.2)	182 (10.3)	< 0.001
RRT any moment	611 (20)	184 (14.4)	427 (24.2)	< 0.001
Exclusive palliative care	354 (12)	124 (9.7)	230 (13.0)	0.006
ICU death	651 (21)	202 (15.8)	449 (25.4)	< 0.001
Main causes of ICU admission				
Shock syndrome	730 (24)	231 (18.1)	499 (28.2)	< 0.001
Respiratory failure	523 (17)	188 (14.7)	335 (19.0)	0.002
High risk post-operative	461 (15)	221 (17.3)	240 (13.6)	0.006
Sepsis	393 (13)	157 (12)	236 (19)	0.548
Acute neurological disorders	177 (6)	87 (6.8)	90 (5.1)	0.255
Others†	1135 (37)	524 (41.0)	611 (34.6)	< 0.001
Comorbidities				
Arterial hypertension	565 (19)	243 (19.0)	322 (18.2)	0.619
Diabetes mellitus	356 (12)	148 (11.6)	208 (11.8)	0.911
AIDS	240 (8)	108 (8.4)	132 (7.5)	0.359
Cirrhosis	218 (7)	99 (7.7)	119 (6.7)	0.321
Metastatic neoplasms	226 (7)	103 (8.1)	123 (7.0)	0.287
Heart failure	180 (6)	73 (5.7)	107 (6.1)	0.746
COPD	196 (6)	91 (7.1)	105 (5.9)	0.220
Atrial fibrillation	179 (6)	82 (6.4)	97 (5.5)	0.322
Oncohematological diseases	114 (4)	47 (3.7)	67 (3.8)	0.943

SBE - standard base excess; SAPS - Simplified Acute Physiological Score; SOFA - Sequential Organ Failure Assessment; pCO_2 - partial pressure of carbon dioxide; ICU - intensive care unit; LOS - length-of-stay; MV - mechanical ventilation; RPT - renal replacement therapy; AIDS - Acquired Immunodeficiency Syndrome; COPD - chronic obstructive pulmonary disease. * p value of the *t*-test or the Chi-squared test, as appropriate, between the groups of acid-base diagnostics. † others are a group of syndromes: polytrauma, cardiogenic shock, severe electrolyte derangements, aortic pathologies, severe dermatologic diseases, burns, digestive hemorrhage, acute and acute on chronic liver failure, severe pancreatitis, diabetic ketoacidosis, severe intoxications and thyroid storm. Results expressed as n, mean ± standard deviation or n (%). n (%) denotes the number of patients and the percentage of the total patients within one group of acid-base diagnostic.

Table 2S - Analysis of survival association of pCO_2 maximum variation of patients categorized according to the intensive care unit length-of-stay

	Model 1 - Basic model with maximum variation of pCO_2 (n = 3,046)		Model 2 - Model 1 with patients with ICU-LOS < 5 days (n = 1,198)		Model 3 - Model 1 with patients with ICU-LOS ≥ 5 days (n = 1,848)	
	OR (95%CI)	p value	OR (95%CI)	p value	OR (95%CI)	p value
Age	1.017 (1.011 - 1.023)	< 0.001	1.022 (1.011 - 1.033)	< 0.001	1.017 (1.010 - 1.024)	< 0.001
SAPS 3	1.042 (1.035 - 1.048)	< 0.001	1.065 (1.053 - 1.078)	< 0.001	1.030 (1.022 - 1.038)	< 0.001
Total SOFA	1.159 (1.130 - 1.189)	< 0.001	1.191 (1.143 - 1.242)	< 0.001	1.139 (1.101 - 1.179)	< 0.001
Modified Charlson	0.957 (0.905 - 1.013)	0.129	0.862 (0.768 - 0.966)	0.010	1.002 (0.939 - 1.070)	0.941
Syndromic diagnosis	0.995 (0.943 - 1.050)	0.859	0.992 (0.895 - 1.099)	0.877	0.991 (0.930 - 1.057)	0.785
SBE	0.973 (0.956 - 0.990)	0.002	0.936 (0.905 - 0.969)	< 0.001	0.989 (0.968 - 1.010)	0.301
Lactate	1.007 (1.003 - 1.012)	0.002	1.014 (1.005 - 1.022)	0.001	1.006 (0.994 - 1.019)	0.417
Maximum pCO_2 variation	1.004 (0.994 -1.013)	0.437	0.997 (0.981 - 1.013)	0.721	1.006 (0.994 - 1.019)	0.329

OR - odds ratio; 95%CI - 95% confidence interval; ICU - intensive care unit; LOS - length-of-stay; SAPS - Simplified Acute Physiological Score; SOFA - Sequential Organ Failure Assessment; SBE - standard base excess. The maximum pCO_2 variation (Vmax) was calculated as the maximum difference between pCO_2 from days 1 to 5 (or up to the last intensive care unit day) subtracted from pCO_2 of the patient's intensive care unit admission. All models were built using binary logistic regression.

Table 3S - Predictors of metabolic acidosis (SBE < - 2 mEq/L) at intensive care unit admission and during intensive care unit stay

	Model 1 - SBE < - 2mE/L at ICU admission clinical predictors (n = 3,046 patients)		Model 2 - SBE < - 2mE/L at ICU admission, clinical and laboratory predictors (n = 3,046 patients)		Model 3 - SBE < - 2mE/L after ICU admission clinical predictors* (n = 1,279 patients)		Model 4 - SBE < - 2mE/L after ICU admission clinical and laboratory predictors* (n= 1,279 patients)	
	OR (95%CI)	p value	OR (95%CI)	p value	OR (95%CI)	p value	OR (95%CI)	p value
Age	0.992 (0.988 - 0.996)	< 0.001	0.992 (0.988 - 0.996)	< 0.001	0.995 (0.989 - 1.002)	0.159	0.996 (0.990 - 1002)	0.228
SAPS 3	1.015 (1.010 - 1.021)	< 0.001	1.012 (1.007 - 1.018)	< 0.001	1005 (0.996 - 1.014)	0.281	1.004 (0.995 - 1013)	0.367
Total SOFA	1.037 (1.014 - 1.060)	0.001	1.027 (1.005 - 1.051)	0.017	1.074 (1.038 - 1.111)	< 0.001	1.077 (1.041 - 1.115)	< 0.001
Modified Charlson	0.986 (0.947 - 1.027)	0.497	0.976 (0.936 - 1.017)	0.249	1.038 (0.976 - 1.105)	0.230	1.030 (0.967 - 1.096)	0.359
Mechanical ventilation	0.945 (0.775 - 1153)	0.578	1.009 (0.824 - 1.236)	0.930	0.629 (0.452 - 0.876)	0.006	0.638 (0.456 - 0.892)	0.008
Maximum heart rate	1.004 (1.000 - 1.007)	0.029	1.004 (1.001 - 1.008)	0.014	0.993 (0.988 - 0.999)	0.022	0.994 (0.988 - 0.999)	0.029
Maximum respiratory rate	0.989 (0.979 - 0.999)	0.024	0.990 (0.981 - 1.000)	0.050	1.000 (0.985 - 1.016)	0.967	0.999 (0.983 - 1.015)	0.862
Maximum temperature	0.932 (0.884 - 0.982)	0.008	0.930 (0.882 - 0.981)	0.008	0.979 (0.897 - 1.068)	0.628	0.977 (0.895 - 1.067)	0.602
Syndromic diagnoses								
Respiratory failure	1.178 (0.960 - 1.445)	0.116	1.088 (0.883 - 1.341)	0.428	0.917 (0.654 - 1.286)	0.616	0.917 (0.652 - 1.289)	0.619
Shock	1.310 (1.070 - 1.604)	0.009	1.251 (1.018 - 1.537)	0.033	0.927 (0.663 - 1.295)	0.655	0.896 (0.640 - 1.255)	0.523
Sepsis	1.054 (0.803 - 1.383)	0.704	1.024 (0.776 - 1.351)	0.868	1.096 (0.713 - 1.683)	0.676	1.084 (0.703 - 1.670)	0.716
Postoperative	0.849 (0.689 – 1.047)	0.126	0.884 (0.715 - 1.094)	0.257	1.101 (0.803 - 1.510)	0.550	1.045 (0.760 - 1.436)	0.787
Neurological	0.801 (0.580 - 1.106)	0.177	0.847 (0.610 - 1.177)	0.323	0.702 (0.423 - 1.166)	0.171	0.682 (0.410 - 1.134)	0.140
Laboratory data								
Admission leukocyte count	----------	--------	1.011 (1.001 - 1.022)	0.026	----------	--------	0.987 (0.968 - 1.006)	0.182
Admission creatinine	----------	--------	1.236 (1.182 - 1.292)	< 0.001	----------	--------	1054 (0.982 - 1.131)	0.144
Admission SBE	----------	--------	----------	--------	----------	--------	0.938 (0.900 - 0.978)	0.002
AUC of the model	0.621 (0.601 - 0.641)	< 0.001	0.665 (0.646 - 0.684)	< 0.001	0.596 (0.564 - 0.629)	< 0.001	0.611 (0.579 - 0.642)	< 0.001

SBE - standard base excess; ICU - intensive care unit; OR - odds ratio; 95%CI - 95% confidence interval. SAPS - Simplified Acute Physiological Score; SOFA - Sequential Organ Failure Assessment; AUC - area under the curve.

* This SBE < - 2mEq/L is a new metabolic acidosis, that is, patients who were admitted to the ICU with SBE ≥ - 2mEq/L and developed a new SBE < - 2mEq/L.

The admission leukocyte count for all patients (N = 3,046) was (median [P25th, P75th]) 10590 [7370,14740] cells/mm³. The admission leukocyte count for patients without metabolic acidosis at admission (N = 1,279) was 10550 [7590,14230] cells/mm³. The admission creatinine for all patients (N = 3046) count was 1.12 [0.76,2.31] mg/dL. The admission creatinine for patients without metabolic acidosis at admission (N = 1,279) was 0.96 [0.70,1.53] mg/dL The admission SBE for all patients (N = 3046) count was -3.6 ± 5.9mEq/L. The admission standard base excess for patients without metabolic acidosis at admission (N = 1,279) was 0.7 ± 3.0mEq/L. All models were built using a binary logistic regression. The hierarchical analysis of adding the laboratory data to the models: Akaike information criteria (AIC) of model 1 was 4032.1, and the Akaike information criteria of model 2 was 3910.6. The comparative analysis of variance of models 1 and 2 resulted in a p-value < 0.001. The AIC of model 3 was 1669.3, and the Akaike information criterion of model 4 was 1658.4. The comparative analysis of variance of models 3 and 4 resulted in a p-value < 0.001.

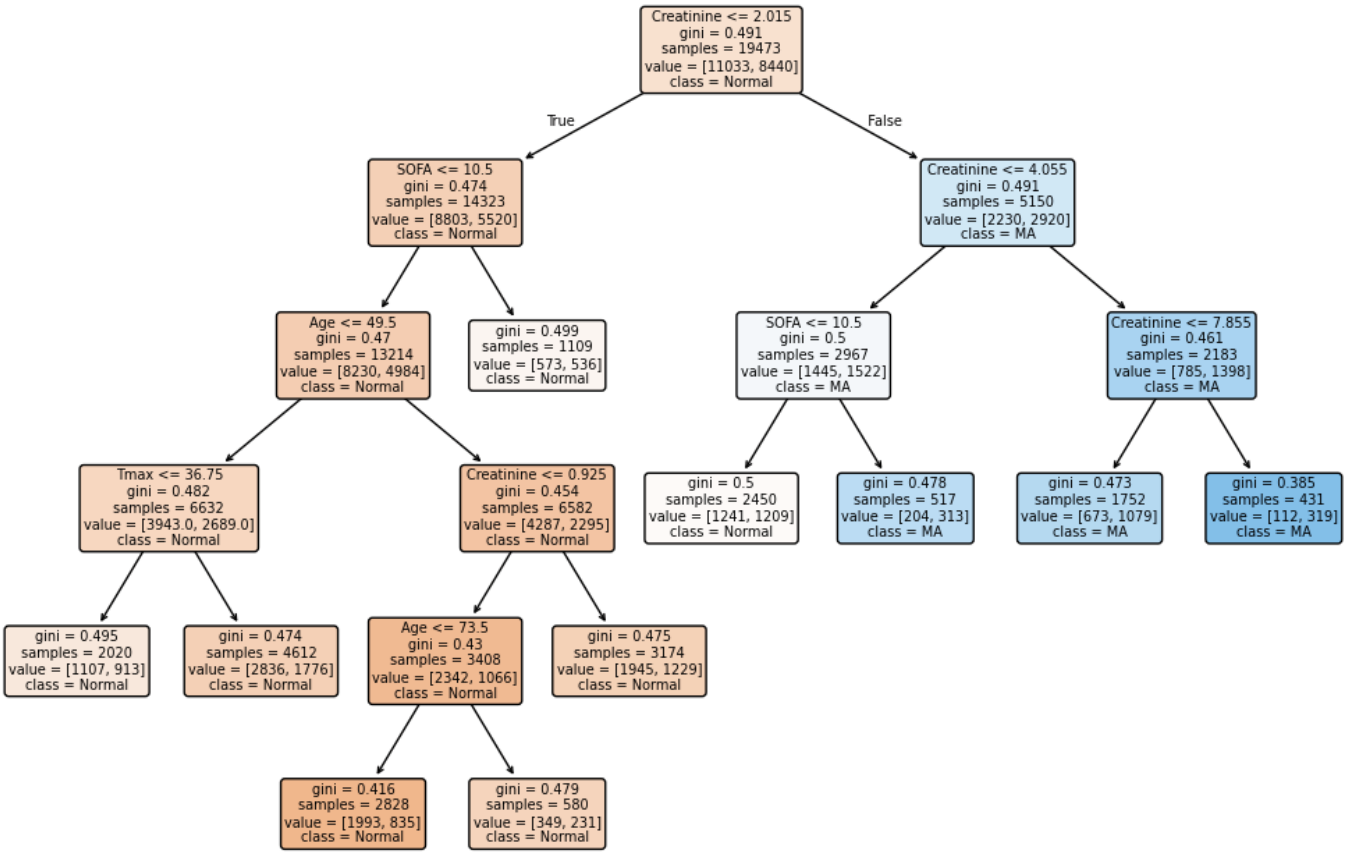

SOFA - Sequential Organ Failure Assessment; MA - metabolic acidosis; Tmax denotes maximum temperature.

Figure 5S - Decision tree from a learning machine analysis of clinical and laboratory predictors of metabolic acidosis at the intensive care unit admission.

Accuracy of the model = 0.62. Sequential Organ Failure Assessment denotes total Sequential Organ Failure Assessment. The variables initially explored were age, Simplified Acute Physiology Score III, total Sequential Organ Failure Assessment, Charlson score, syndromic diagnosis, maximum heart rate, maximum respiratory rate, maximum temperature, and mechanical ventilation need. Class is the presence or absence of the diagnosis of metabolic acidosis. The sample is the number of patients in each node. Value is the number of patients without and diagnosed with metabolic acidosis, respectively. Orange color with lesser transparency shows the nodes with a higher probability of the absence of diagnosis of metabolic acidosis. Blue color with lesser transparency shows the nodes with higher probability of diagnosing metabolic acidosis. Gini index denotes the inhomogeneity of the patient's sample of the node. The less the Gini index higher the probability of a given diagnosis at that node.
